# A novel approach for managing the incisions of tibial plateau fractures with soft tissue swelling

**DOI:** 10.1038/s41598-025-86125-5

**Published:** 2025-01-21

**Authors:** Yang Zhang, Yangyang Zhao, Shuanggong Liu, Zihan Yao, Zikang Jin, Wanli Ma

**Affiliations:** 1 Department of Orthopaedics, The Second Hospital, Cheeloo College of Medicine, Shandong University, 247 Beiyuan Street, Jinan, 250033 Shandong People’s Republic of China; 2 Department of Orthopaedics, Linglong Yingcheng Hospital, Zhaoyuan, 264000 Shandong People’s Republic of China

**Keywords:** Tibial plateau fractures, Soft tissue damage, Incision management, Clinical efficiency, Outcomes research, Clinical trial design, Trauma

## Abstract

To investigate the feasibility and clinical efficacy of a novel approach to managing the incisions used to treat tibial plateau fractures (TPFs) with soft tissue swelling. We retrospectively enrolled 64 patients with TPFs who underwent surgery at the Second Hospital of Shandong University. Patients were divided into two groups: Group A (*n* = 32) underwent early surgery with the novel incision management technique, and Group B (*n* = 32) underwent conventional surgery after swelling reduction. The perioperative data of the two groups were compared, including the time from injury to surgery, one-stage operation time, intraoperative blood loss, number of dressing changes, wound healing time, and hospitalization time. Preoperative and postoperative complications were assessed in both groups, and pain condition, degree of arthritis, limb function, imaging results, and quality of life were evaluated using validated scales. The time from injury to surgery, number of dressing changes, and hospitalization time in Group A were significantly lower than those in Group B (*P* < 0.05). There were no significant differences in one-stage operation time, intraoperative blood loss, or wound healing time between the two groups (*P* > 0.05). There were fewer preoperative and postoperative complications in Group A than in Group B (*P* < 0.05). The VAS and WOMAC scores were reduced in both groups (*P* < 0.05); Group A had lower VAS scores two weeks after surgery. There was no statistically significant difference in the WOMAC score between the groups. The modified Rasmussen functional and radiological scores were elevated in both groups (*P* < 0.05). There was no statistically significant difference between the two groups for the modified Rasmussen functional or radiological score at any of the time points (*P* > 0.05). In addition, the two groups did not differ in quality of life (*P* > 0.05). For patients with tibial plateau fractures without congestive blisters, open reduction and internal fixation at the early stage of swelling and wide-spacing interrupted suture and negative pressure wound therapy (NPWT) closure of the wound could help obtain excellent to good functional outcomes with fewer complications. This novel incision management approach and concept expands the surgical indications for these fractures.

**Supplementary Information:**

The online version contains supplementary material available at 10.1038/s41598-025-86125-5.

## Introduction

Tibial plateau fractures (TPFs) are complex injuries caused by direct trauma or indirect compressive forces, and the severity of these fractures ranges from nondisplaced to complicated^1^. Clinical data suggest that the incidence of TPFs is approximately 1% of all fractures and 8% of fractures in older people; their increasing frequency and severity of complications present severe challenges for orthopaedists^2,3^. In recent years, surgical treatments for TPFs have been developed, but traditional open reduction internal fixation (ORIF) is still the gold standard for their treatment. In addition, TPFs are often accompanied by associated soft tissue lesions that can affect their surgical indications. For complex fracture types, sequential (staged) treatment (external fixation with subsequent definitive ORIF at a later stage) is the standard approach^4,5^. Early fracture reduction, articular surface repair, and knee functional exercise as early as possible are the main objectives of surgical treatment for TPFs^6^. Some joint injuries can be treated with minimally invasive methods such as arthroscopy to assist in fracture reduction and repair of intraarticular soft tissue injuries. It is important to note that soft tissue complications can occur with any tibial plateau fracture^7^.

In TPFs, the extent of soft tissue damage around the knee joint is critical. Traumatic fractures can damage surrounding tissues, triggering an inflammatory response and swelling. Oedema and haematoma increase pressure within the fascial compartment, compromising the blood supply, exacerbating muscle ischaemia and hypoxia, releasing more metabolic byproducts, and inducing further inflammation and oedema, leading to a progressively worsening vicious cycle and ultimately resulting in tissue necrosis and nerve dysfunction^8^. When the compartment pressure surpasses the tensile threshold of the tissue, separation occurs between the epidermis and dermis, resulting in fluid accumulation and the formation of tension blisters. Compared to clear fluid-filled blisters, blood-filled blisters indicate a loss of vitality in the epidermal layer, even suggesting the possibility of severe necrosis in the deep dermis and muscle, which can increase the risk of poor surgical wound healing^9,10^. Further aggravation of the swelling may lead to compartment syndrome, and early fasciotomy is necessary to prevent nerve and vascular damage as well as muscle necrosis, followed by staged fracture fixation^11,12^.

**Fig. 1:**
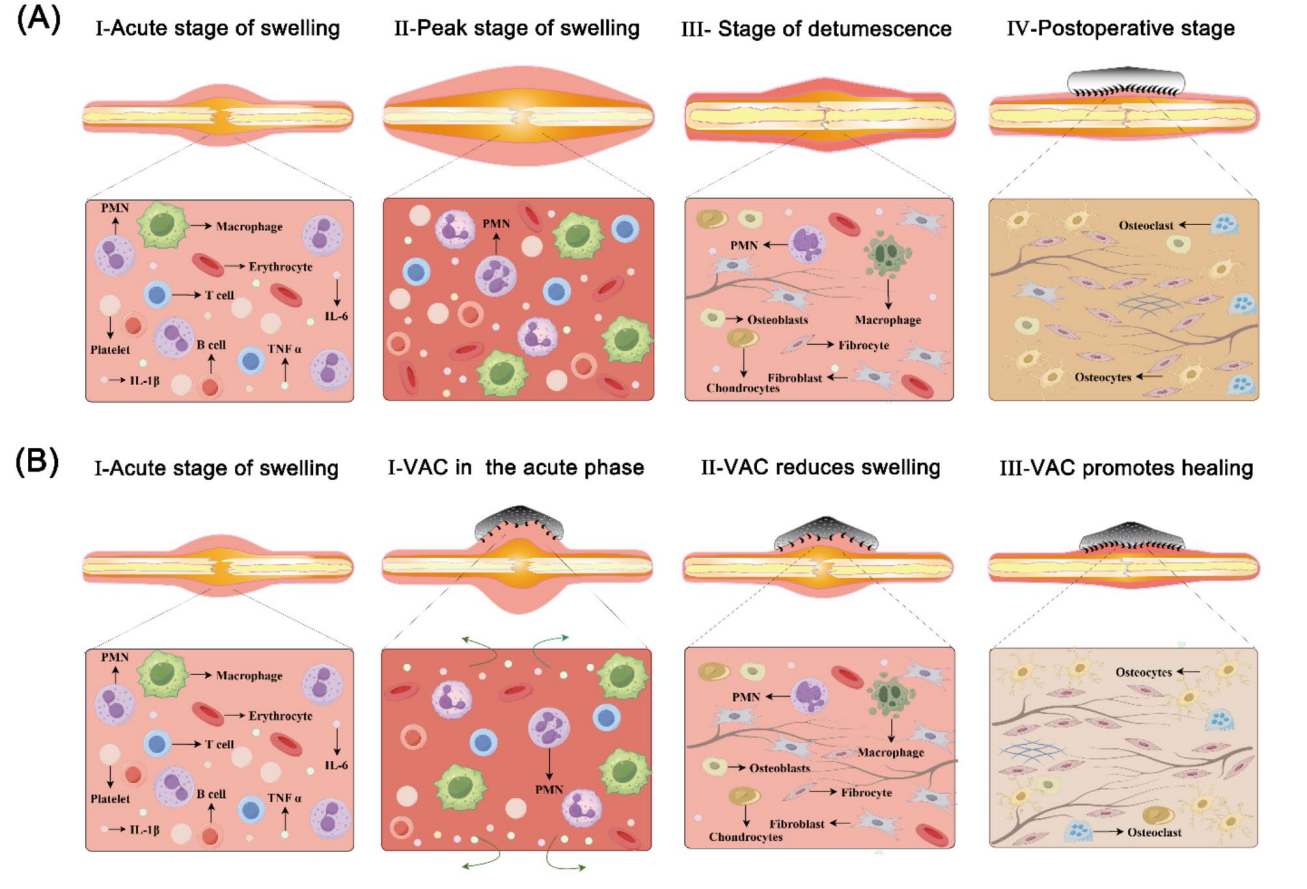
(**A**) Schematic diagram of the histopathological process of the traditional surgical method. (**B**) Schematic diagram of the histopathological process of the novel wound management strategy.

Traditional concepts and the literature worldwide support the consensus that surgery should only be performed after the swelling subsides to prevent serious consequences such as soft tissue necrosis, wound dehiscence, and exposed plates (Fig. 1A)^13,14^. The waiting period for swelling reduction and staged surgery significantly prolongs the treatment duration and is associated with various complications, as well as increasing the financial burden on patients and society. However, no technology or concept is currently available to circumvent this issue. Negative pressure wound therapy (NPWT) has become an important treatment modality for various types of wounds and is suitable for the treatment of surgical wound dehiscence, deep pressure injury, diabetic foot ulcer, infection, limb soft tissue injury, open fracture, and skin grafting^15,16^. NPWT provides a closed, sterile, and relatively moist environment for wound tissue and is conducive to granulation tissue proliferation. Simultaneously, it accelerates local blood microcirculation, promotes drainage, prevents infection, and creates favourable conditions for wound healing^17^.

In this study, we applied a novel wound management strategy for the treatment of tibial plateau fractures with soft tissue swelling, aiming to investigate the feasibility and clinical efficacy of this approach. Our objective was to provide orthopedic surgeons with new perspectives and evidence to support clinical decision-making. For TPFs without congestive blisters, we hypothesized that the use of open reduction and internal fixation in the early stage of swelling, followed by wide-spacing interrupted sutures and NPWT to close the wound, would accelerate swelling reduction, promote wound healing, and avoid adverse outcomes (Fig. 1B). We describe our novel early surgical wound management techniques and concepts in this setting, which expand the surgical indications for TPFs with soft tissue swelling compared to conventional wisdom. We compared the new wound management model with traditional surgery regarding perioperative indices, joint function and imaging efficacy, preoperative and postoperative complications, and quality of life, providing new ideas and a basis for clinical surgical decision-making.

## Materials and methods

Our research received approval from the Ethics Committee of the Second Hospital of Shandong University (approval number: KYLL2024455). All protocols were conducted according to the relevant national ethical guidelines and the principles outlined in the Helsinki Declaration. Additionally, written informed consent was obtained from all participants (or their legal guardians) prior to their inclusion in the study.

### Study characteristics

**Fig. 2:**
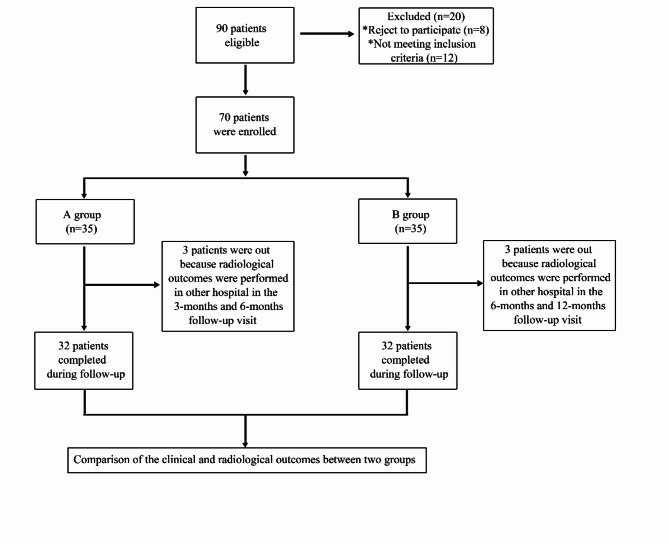
The flow of participants included in this study.

**Fig. 3:**
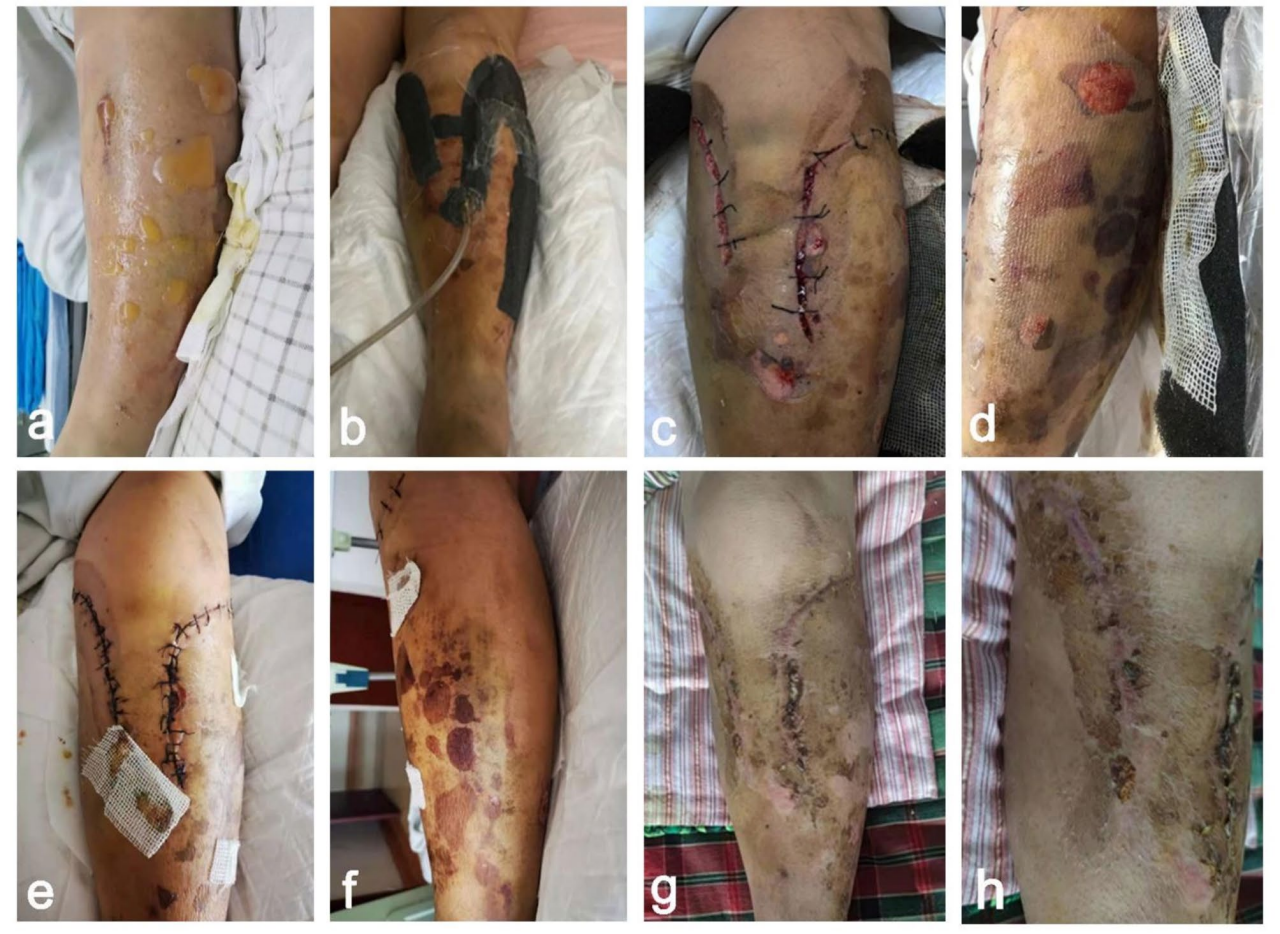
(**a**) Serous tension blisters appeared on the skin before surgery. (**b**) The incision was closed by the NPWT. (**c**,**d**) After three days, the NPWT dressing was replaced, oedema was reduced, the skin margin was relaxed, and a secondary suture was performed. (**e**,**f**) The incision was sutured routinely after the NPWT dressing was removed. (**g**,**h**) The incision was scabbed and healed well.

**Fig. 4:**
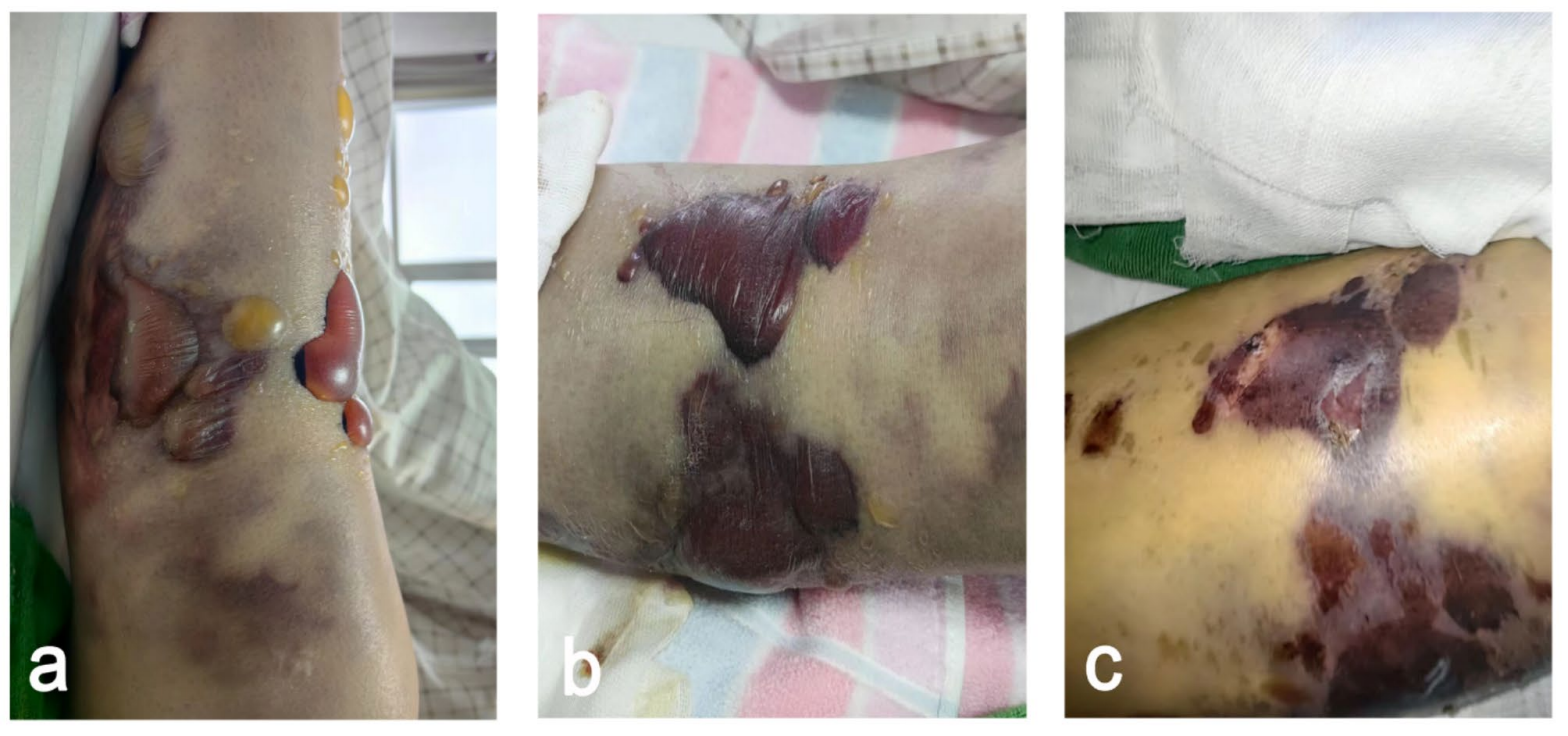
(**a**–**c**) Illustration of bloody blisters; the appearance is dark red and cloudy, with a spot indicating a bleeding area.

We retrospectively included patients with TPFs admitted to the Second Hospital of Shandong University between February 2020 and December 2022. Patients were categorized into two groups. Group A (32 patients) underwent novel incision management, and Group B (32 patients) underwent conventional suturing, as shown in Fig. 2. The inclusion criteria were as follows: age ≥ 18 years, indirect trauma, a fresh fracture within two weeks from injury, soft tissue swelling grade II or III (Supplementary Table 1; Fig. 3a), tolerance of surgery, and complete data availability until follow-up. The exclusion criteria were as follows: direct trauma, surgical contraindications such as severe organ dysfunction, previous tibial surgery, open fractures, pathological fractures, fragility fractures, swelling grade > III or bloody blisters (Fig. 4a–c), incomplete data at follow-up and minimally cooperative patients. Before surgery, the study personnel briefed the patients and their families on the two surgical procedures. The choice of procedure was made based on the patient’s soft tissue status and doctor‒patient communication. The surgical team then performed the surgery using the selected incision treatment approach.

### Preoperative preparation

**Fig. 5:**
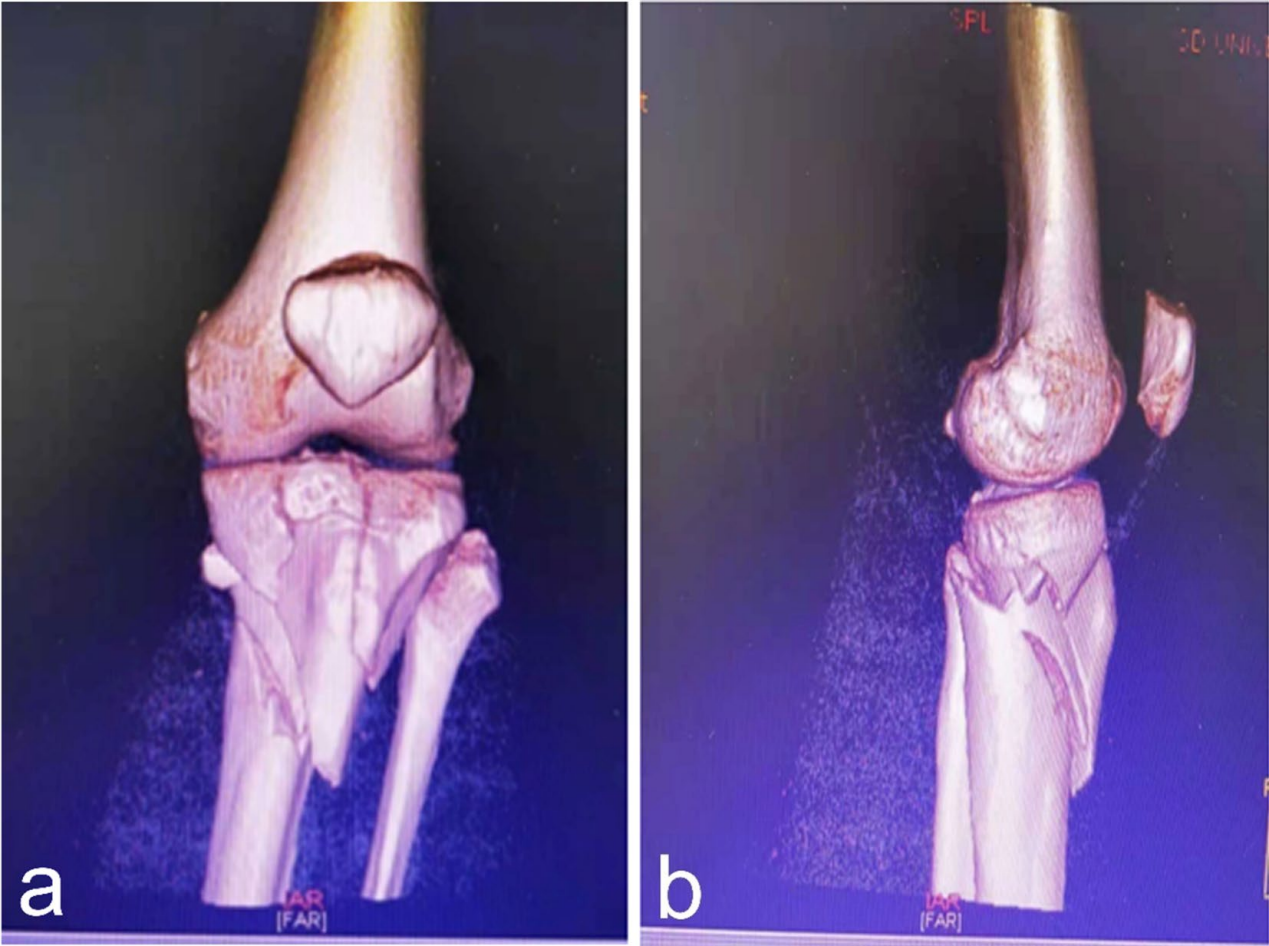
(**a**,**b**) CT 3D reconstruction scan for preoperative planning.

All patients were treated with anti-inflammatory and analgesic medications upon admission. Preoperative radiographs in two planes were taken for all patients. A computed tomography (CT) scan with three-dimensional reconstruction was used to better understand the fracture configuration and determine the severity of the fracture (Fig. 5a,b). Prophylactic antibiotics were administered to all patients, and appropriate antithrombotic measures were implemented during the waiting period to reduce swelling. All surgeries were conducted at the same clinical center under consistent conditions. After a proper diagnosis, the therapeutic interventions were performed by the same group of trauma surgeons in our department, and the chief surgeon (Dr. Ma) was a senior surgeon, with more than 15 years of experience in a provincial third-class hospital.

### Surgical procedures

Group A: Surgery was performed immediately after completing the haematological and imaging tests. Under spinal anaesthesia, local preparation and suspension were performed without the use of tourniquets. The patient was placed in a supine position, and a longitudinal incision of approximately 15 cm was made on the affected side of the knee joint. The skin, subcutaneous tissue, and deep fascia were cut layer by layer and separated layer by layer to reveal the tibial plateau on the affected side. The fracture was investigated, and the articular surface was reduced to flat with an apical rod along the fracture line. Artificial bone or autogenous iliac bone was implanted when bone loss occurred at the fracture end. Implant bone plate and screw fixation were applied as needed. A C-arm X-ray machine was used to confirm that the fracture reduction effect was satisfactory. After the wound was washed and haemostasis was complete, the wound was treated with a wide-spacing intermittent suture and then covered with a NPWT device. Three to five days postoperatively, the NPWT device was replaced, and the wound was sutured conventionally based on the patient’s wound healing status. The perioperative status of the patients was shown in Fig. 3a–h.

Group B: Ice was applied to the affected limb for 7 ~ 10 days, routine traction was applied, haematology and imaging examinations were performed on all patients, and surgery was performed after eliminating the oedema. Under spinal anaesthesia, local preparation and suspension were performed without the use of tourniquets. The patient was placed in a supine position, and a longitudinal incision of approximately 15 cm was made on the affected side of the knee joint. The skin, subcutaneous tissue, and deep fascia were cut layer by layer and separated layer by layer to reveal the tibial plateau on the affected side. The fracture was investigated, and the articular surface was reduced to flat with an apical rod along the fracture line. Artificial bone or autogenous iliac bone was implanted when bone loss occurred at the fracture end. Implant bone plate and screw fixation were applied as needed. A C-arm X-ray machine was used to confirm that the fracture reduction effect was satisfactory. The wound was then irrigated, electrocoagulation was used to stop the bleeding, the wound was sutured sequentially, and the wound was bandaged with sterile adjuvant.

**Fig. 6:**
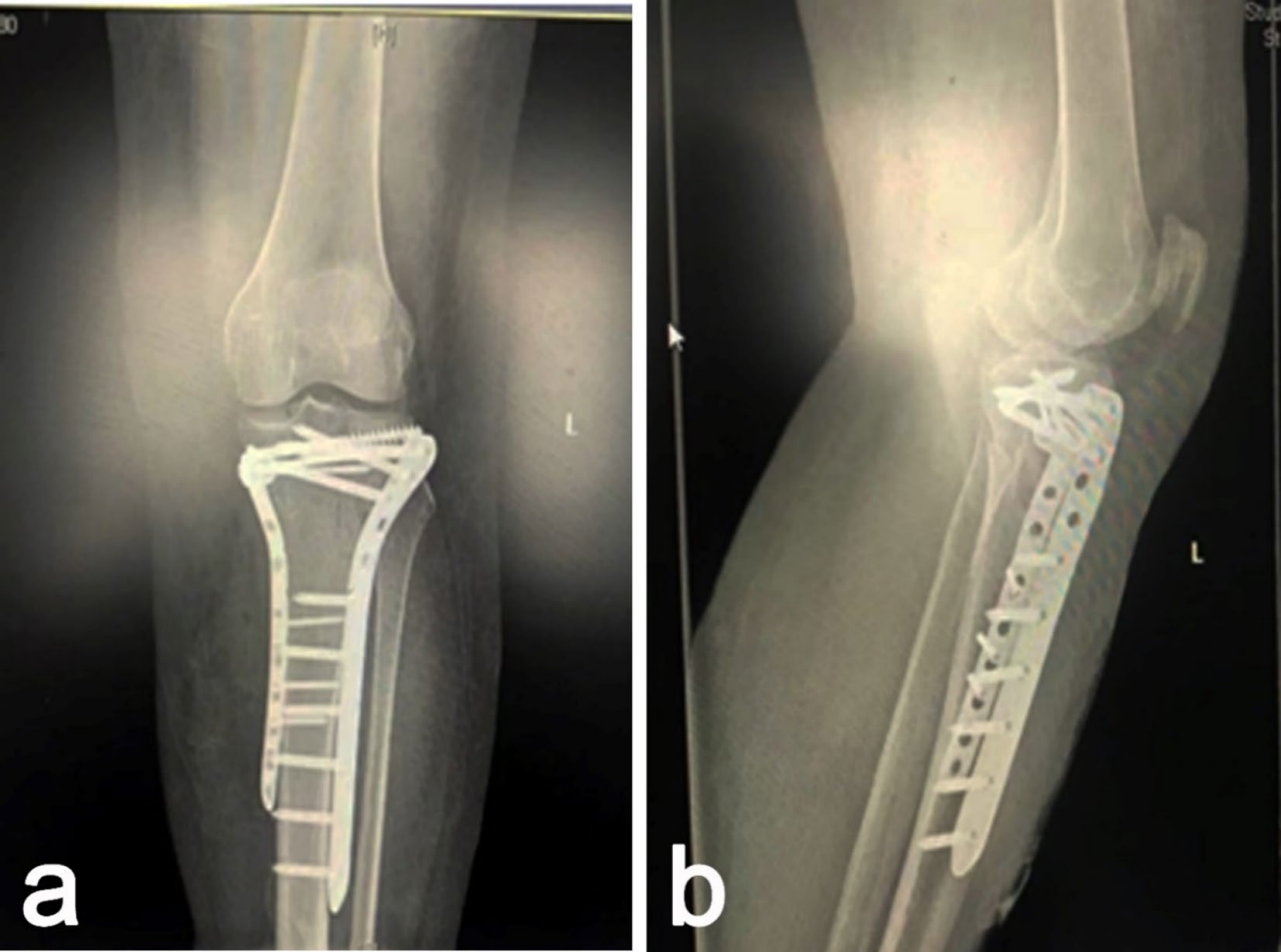
Postoperative X-rays showed that the fracture was well fixed.

### Postoperative management

All patients received guided rehabilitation training with conventional anti-infective and analgesic drugs. Patients in Group A underwent a second surgery to replace the NPWT dressing three days after the initial surgery and the incision was closed with traditional sutures. In contrast, patients in Group B underwent regular dressing changes according to the condition of wound leakage, which was routinely changed every two days until 14 days, when the sutures were removed. All patients underwent postoperative X-ray examination 1–2 days postoperatively to assess fracture reduction, evaluate the position and stability of internal fixation devices (Fig. 6a,b).

### Outcome measures

Preoperative complications, including deep vein thrombosis, pulmonary infections, and decubital ulcers, were recorded and compared between the two groups. The same investigator collected perioperative data such as the time from injury to surgery, one-stage operation time, intraoperative blood loss, number of dressing changes, postoperative bed time and hospitalization time. Postoperative complications, including superficial infection, deep infection, deep vein thrombosis, hypostatic pneumonia, decubital ulcer, neurologic pain, wound dehiscence, nonunion, lysis of adhesions, and osteomyelitis, were recorded and compared between the two groups. The visual analogue scale (VAS) and Western Ontario and McMaster University Osteoarthritis Index (WOMAC) were used to assess pain and the degree of arthritis at four time points (2 weeks after surgery, three months after surgery, six months after surgery, and 12 months)^18^. The modified Rasmussen functional and radiological scoring (Supplementary Tables 2 and 3) was used to assess functional and radiological outcomes at four time points (before surgery, three months after surgery, six months after surgery, and 12 months). The 36-Item Short-Form Health Survey (SF-36) was used to assess quality of life 12 months after surgery. The SF-36 consists of eight attributes: physical function (PF), role-physical (RP), body pain (BP), general health (GH), vitality (VT), role-emotional (RE), and mental health (MH). The total score for each section is 100, with a lower score indicating lower quality of life^19^.

### Statistical analysis

The data were analysed using SPSS software (version 26.0) and GraphPad Prism 9 software. Continuous variables are described as the mean ± standard deviation. According to the normal distribution of the data, we used the t test to compare means between groups. Intragroup time point comparisons were performed using single factor repeated-measures ANOVA, and pairwise comparisons were performed using the least significant difference (LSD) method. When the data were not normally distributed, we used the rank-sum test. We used the Pearson chi-square test (χ2 test) and Fisher’s exact test to analyse categorical variables. The Mann‒Whitney U test was used to assess grade data, and intragroup comparisons were made using the Friedman test with multiple relevant data checks. A *p* value < 0.05 was considered to indicate statistical significance.

## Results

### Baseline characteristics

We included 64 patients with bicondylar TPFs (32 patients in each group). Among them, 26 were women and 38 were men. The average age was 46.31 ± 15.12 years. The demographic variables included age, sex, BMI, injury time, follow-up time, loss to follow-up rate, and comorbidities, including hypertension, diabetes, cardiopathy, hyperlipidaemia, smoking status, alcohol status, and bone quality. The procedure-related variables included ASA classification, Schatzker type, and Tscherne grade for closed fractures at presentation. Table 1 shows that the baseline characteristics did not significantly differ among the groups (*p* > 0.05).

**Table 1:** Baseline characteristics (n and %).

	*A (n = 32)*	*B (n = 32)*	t/X^2^/Z	*p* value
Sex			0.259	0.611
Male	20	18		
Female	12	14		
Age (years)	45.47 ± 14.16	47.14 ± 16.07	0.463	0.645
BMI (kg/m^2^)	23.11 ± 1.48	24.59 ± 1.76	0.927	0.741
Injury time (hours)	4.76 ± 2.12	5.35 ± 2.34	0.874	0.369
Schatzker type			-0.335	0.738
1	1 (3.12)	3 (9.38)		
2	20 (62.50)	16 (50.00)		
3	3 (9.38)	1 (3.12)		
4	5 (15.63)	7 (21.88)		
5	1 (3.12)	4 (12.50)		
6	2 (6.25)	1 (3.12)		
Tscherne grade			− 0.1.160	0.246
C0	13 (40.62)	17 (53.13)		
C1	16 (50.00)	14 (43.75)		
C2	3 (9.38)	1 (3.12)		
Comorbidities				
Hypertension	11 (34.38)	14 (43.75)	0.591	0.442
Diabetes	7 (21.88)	9 (28.12)	0.333	0.564
Cardiopathy	6 (18.75)	4 (12.50)	0.474	0.491
Hyperlipidaemia	9 (28.12)	6 (18.75)	0.784	0.376
ASA classification			-0.691	0.490
1	8 (25.00)	9 (28.12)		
2	15 (46.87)	17 (53.13)		
3	6 (18.75)	4 (12.50)		
4	3 (9.38)	2 (6.25)		
Smoking status			1.168	0.558
Current	11 (34.38)	12 (37.50)		
Former	6 (18.75)	3 (9.38)		
Never	15 (46.87)	17 (53.12)		
Alcohol status			0.644	0.725
Current	16 (50.00)	14 (43.75)		
Former	3 (9.38)	2 (6.25)		
Never	13 (40.62)	16 (50.00)		
Bone quality				
Osteoporosis	1 (3.12)	3 (6.25)		0.613
Osteopenia	3 (9.38)	5 (15.63)		0.708
Follow-up time	12.24 ± 1.74	13 ± 1.32	1.026	0.624
Lost to follow-up	1 (3.12)	2 (6.25)	0.350	0.554

### Preoperative complications

Before surgery, the B group experienced several complications, including deep vein thrombosis (*n* = 3), pulmonary infections (*n* = 2), and decubital ulcers (*n* = 2). The complication rate was 21.88%. In Group A, none of these complications occurred. Although there was no significant difference in any single complication between the two groups, the overall complication rate in Group A was significantly lower than that in Group B (0.00% vs. 21.88%, *P* < 0.05). The results are displayed in Table 2.

**Table 2:** Preoperative complications (n and %).

	*A (n = 32)*	*B (n = 32)*	X^2^	*p* value
Deep vein thrombosis	0 (-)	3 (9.38)	-	0.238
Pulmonary infection	0 (-)	2 (6.25)	-	0.492
Decubital ulcer	0 (-)	2 (6.25)	-	0.492
Complication rate	0 (-)	7 (21.88)	-	0.011

### Comparisons of perioperative indices

**Fig. 7:**
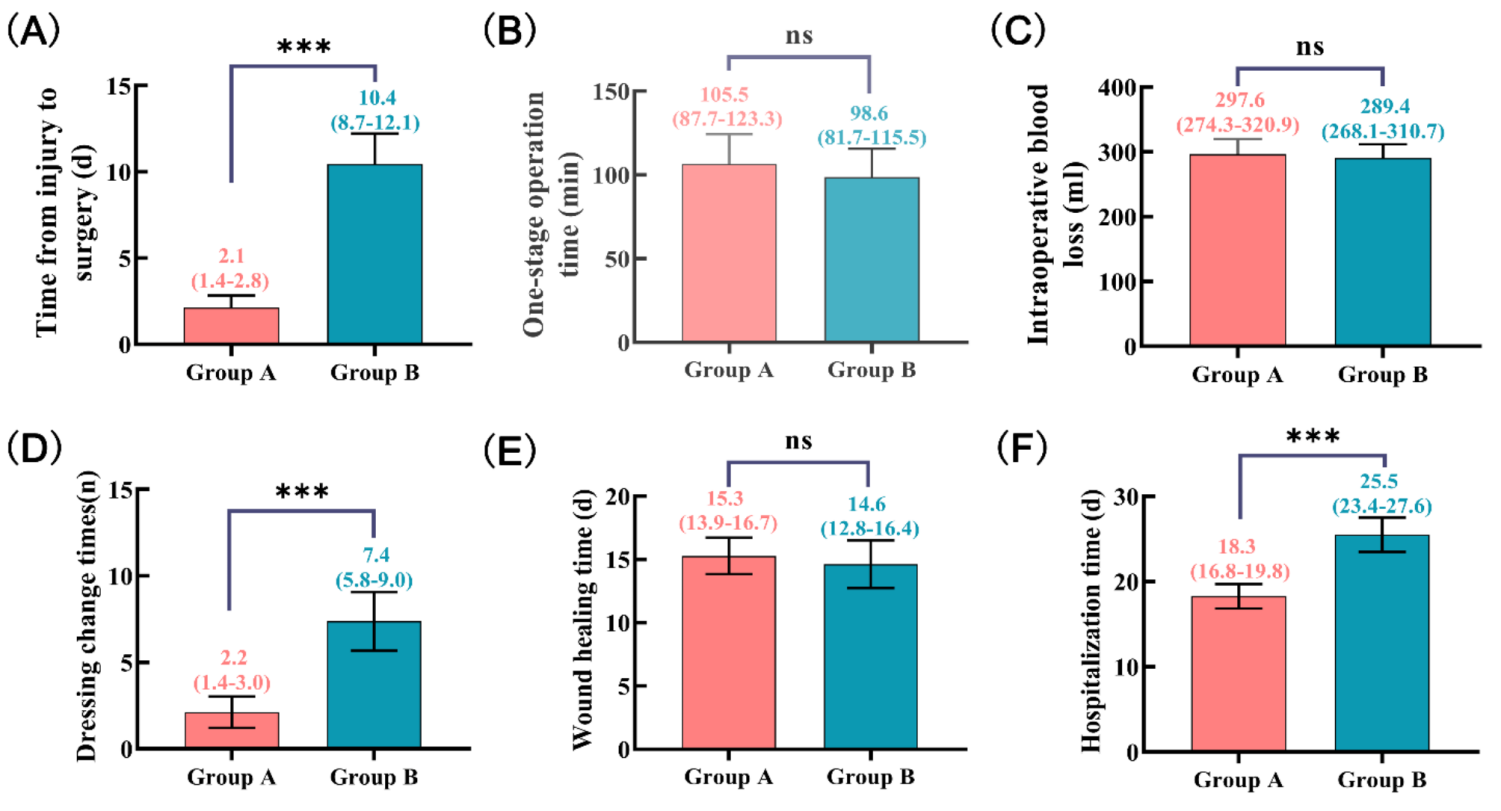
Perioperative data (**A**) Time from injury to surgery. (**B**) One-stage operation time. (**C**) Intraoperative blood loss. (**D**) Dressing change times. (**E**) Wound healing time. (**F**) Hospitalization time. *p < 0.05. **p < 0.001.

In terms of the time from injury to surgery, the number of dressing changes and hospitalization time, Group A had significantly fewer dressing changes than did Group B (*P* < 0.05). There were no significant differences in one-stage operation time, intraoperative blood loss, or wound healing time between Group A and Group B (*P* > 0.05). The results are visualized in Fig. 7.

### Comparison of postoperative complications

After treatment, Group A only experienced one case of a deep venous thrombosis (*n* = 1) and no other complications, including no cases of superficial infection, deep infection, hypostatic pneumonia, decubital ulcer, neurologic pain, wound dehiscence, nonunion, lysis of adhesions or osteomyelitis. The complication rate was 3.12%. In Group B, the complications included superficial infection (*n* = 2), deep venous thrombosis (*n* = 3), hypostatic pneumonia (*n* = 2), and wound dehiscence (*n* = 2); two of the patients with wound dehiscence healed after dressing change debridement and suturing. No patients had deep infection, decubital ulcer, neurological pain, nonunion, lysis of adhesions, or osteomyelitis. The complication rate was 28.12%. The complication rate in Group A was lower than that in Group B (3.12% vs. 28.12%, *P* < 0.05). The results are summarized in Table 3.

**Table 3:** Postoperative adverse reactions (n and %).

	*A (n = 32)*	*B (n = 32)*	X^2^	*p* value
Superficial infection	0	2 (6.25)	-	0.492
Deep infection	0	0	-	–
Deep vein thrombosis	1 (3,12)	3 (9.38)	–	0.613
Hypostatic pneumonia	0	2 (6.25)		0.492
Decubital ulcer	0	0	–	–
Neurologic pain	0	0	–	–
Wound dehiscence	0	2 (6.25)	–	0.492
Nonunion	0	0	–	
Lysis of adhesions	0	0	–	–
Osteomyelitis	0	0	–	–
Complication rate	1 (3.12)	9 (28.12)	–	0.006

### Comparison of VAS function and WOMAC scores

**Fig. 8:**
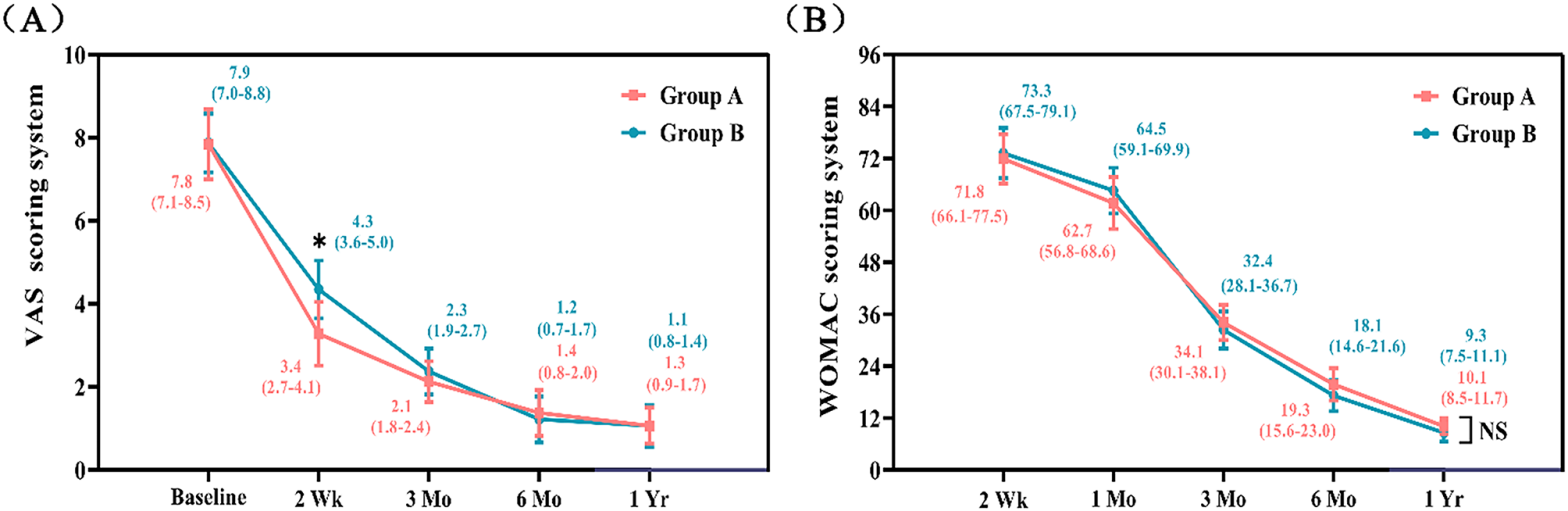
(**A**) VAS scores of the two groups at different time points. (**B**) WOMAC scores of the two groups at different time points. *p < 0.05, ^NS^*p* > 0.05.

Compared with the preoperative scores, the postoperative VAS and WOMAC scores were significantly lower in the two groups (*p* < 0.05). Group A reported lower VAS scores two weeks after surgery (*p* < 0.05), without any difference between the two groups at any other time points (*p* > 0.05). At various time intervals, no statistically significant differences were observed between the groups concerning WOMAC scores (*P* > 0.05). The results are displayed in Fig. 8.

### Comparison of limb function and reduction effects

**Fig. 9:**
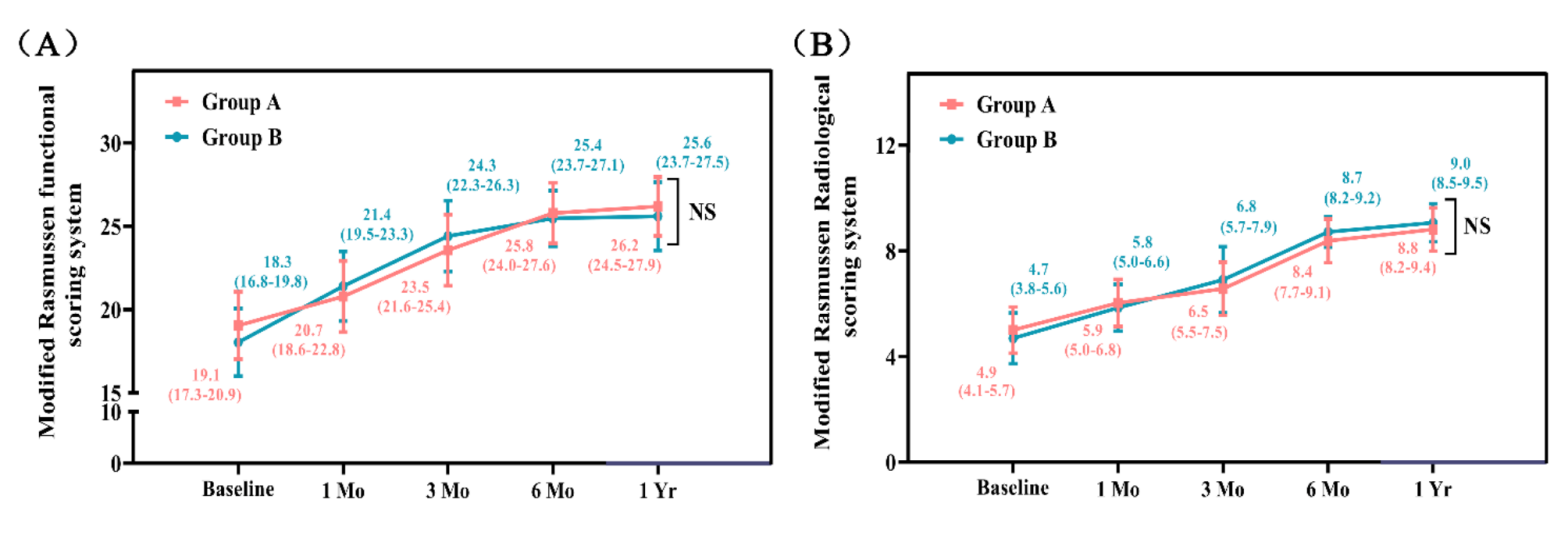
(**A**) Modified Rasmussen functional scoring system for the two groups at different time points. (**B**) Modified Rasmussen radiological scoring system for the two groups at different time points. *p < 0.05, ^NS^*p* > 0.05.

Compared with the preoperative scores, the postoperative modified Rasmussen functional and radiological scores were significantly greater in the two groups (*P* < 0.05). There were no statistically significant difference between the groups in terms of the modified Rasmussen functional or radiological score at any of the time points (*P* > 0.05). The results are visualized in Fig. 9.

### Comparison of quality of life

**Fig. 10:**
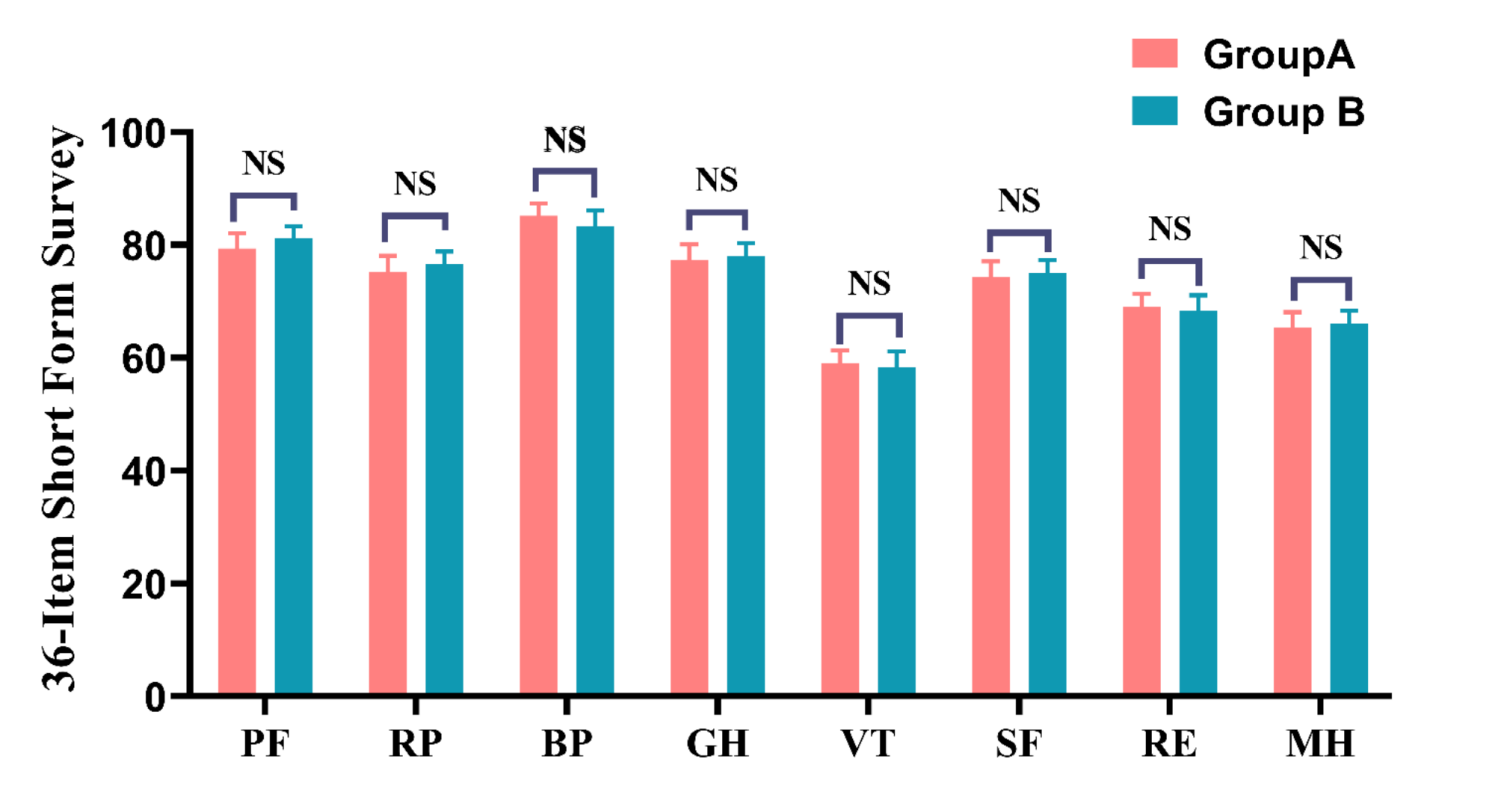
Comparison of the SF-36 score at 12 months postoperatively between the A and B groups. *p < 0.05, ^NS^*p* > 0.05 (paired *t* test).

As illustrated in Fig. 10, one year after surgery, there were no significant differences between the two groups in 8 aspects, namely, physical function (PF), role-physical (RP), body pain (BP), general health (GH), vitality (VT), social function (SF), role-emotional (RE), and mental health (MH) (*p* > 0.05).

## Discussion

TPFs are complex injuries resulting from high-energy or low-energy trauma that present with various morphological patterns. In most cases, they are associated with extensive soft tissue damage, comminuted and highly displaced intra-articular fractures, and joint instability, which can compromise lower limb viability and require complex and comprehensive treatment^20,21^. It is reasonable to assume that the fractures remain constant after the injury while the soft tissue undergoes alterations. Inflammatory responses and soft tissue swelling caused by trauma can delay the timing of surgery, while the occurrence of compartment syndrome necessitates an emergency fasciotomy followed by staged surgical treatment. These traditional concepts and staged procedures are associated with high infection rates, prolonged hospital stays, perioperative adverse events, and increased socioeconomic burdens on patients and society^22^. While ORIF of TPF has shown promising outcomes, it also has notable drawbacks, such as infection, nonunion, pain, stiffness, and posttraumatic arthritis^23–25^. Inflammatory oedema and necrosis of soft tissues may be the primary causes of these complications. A study on ORIF techniques revealed that despite satisfactory functional outcomes, 20% of patients experienced superficial or deep infections^26^. It has been reported that up to 50% of complications are related to soft tissue issues^27^. Therefore, we propose a novel wound management approach that combines ORIF with intermittent suturing and NPWT wound closure. This method not only changes the indications for acute-phase surgery but also effectively prevents various perioperative complications. Additionally, it results in favourable outcomes in terms of limb function, reduction effect and quality of life.

Swelling and deswelling after fracture are complex physiological processes that involve many pathophysiological mechanisms. Posttraumatic oedema can result from an inflammatory response, chemotaxis of immune cells, lymphatic obstruction, deep vein thrombosis, or overactivity of growth factors and cytokines at the trauma site, with swelling usually peaking 4 ~ 5 days after the fracture^28^. With the excretion of these inflammatory factors, the metabolism of the lymphatic system, the formation of new blood vessels, the repair of tissues, and the reabsorption of interstitial fluid, the oedema gradually resolves, usually within 7 ~ 10 days after the traumatic event that caused the fracture^29,30^. Oedema and inflammation associated with trauma can easily lead to local vein damage, dermal hypoxia, and other soft tissue damage^31^. This often leads to blistering of the skin and, in some cases, dermal and even muscle necrosis^32^. Notably, blood-filled blisters indicate severe ischaemia of the dermis and are associated with a poor prognosis. Therefore, any form of surgical intervention is not advisable in these cases. Many studies have reported that vacuum-assisted wound management is effective in hastening the resolution of wound oedema and enhancing local blood flow, which makes it useful in the treatment of osteofascial compartment syndrome^33,34^. Studies have also shown that the application of NPWT can significantly reduce postoperative flap oedema improve arterial macroflow, and improve venous outflow^35^. In addition, NPWT can also be used as a simple and effective treatment option for some lymphatic system diseases^36^. We envision the use of NPWT after TPF to accelerate the natural deswelling process and prevent soft tissue-related complications, and these studies provide a theoretical basis for our new approach. The widely recognized mechanisms of action underlying the therapeutic effect of NPWT include macrodeformation, drainage of fluids, stabilization of the wound environment, and microdeformation^37^. NPWT promotes wound healing by modulating cell signaling mediated by anti-inflammatory factors, mechanoreceptors, and chemoreceptors^38^. It also facilitates angiogenesis, extracellular matrix remodeling, and granulation tissue deposition^39^.

Compared to traditional surgical methods and concepts, our proposed new wound management approach has certain advantages. The preoperative waiting time, frequency of dressing changes, and length of hospital stay in the new technology group were lower than those in the traditional surgery group, significantly reducing the treatment duration and economic burden on patients. In terms of adverse effects, the incidence of preoperative complications in the new technology group was significantly lower than that in the traditional surgery group. Traditional surgical methods require fracture fixation surgery only after soft tissue swelling has subsided, and prolonged preoperative bed rest increases the risk of deep vein thrombosis in the lower limbs, hypostatic pneumonia, and even pressure sores. In contrast, the concept of one-stage surgery with the new technology completely avoids any preoperative complications. Additionally, the incidence of postoperative complications in patients treated with the new technique was significantly lower than that in patients in the traditional surgery group, with no occurrences of soft tissue infection or wound dehiscence.

Regarding VAS scores, patients treated with the new technology reported significantly better improvement at two weeks postoperatively than did those treated with traditional surgery. In addition to surgical factors, inflammatory factors are crucial in the pathogenesis of posttraumatic osteoarthritis^40–42^. The impact of performing surgery during the inflammatory response phase on the development of posttraumatic osteoarthritis is also an important issue to consider. We found no significant differences in WOMAC scores between the two groups at various time points. Multiple studies have shown that NPWT exerts anti-inflammatory effects by removing inflammatory exudate, modulating cytokines to an anti-inflammatory profile, and improving the systemic distribution of these factors^38,43^. Moreover, we also found no significant differences in modified Rasmussen functional or radiological scores between the two groups at different time points. If the soft tissue is highly swollen, reduction and fixation can be more challenging. However, NPWT can help reduce swelling, providing better and more stable soft tissue conditions. In terms of quality of life, both groups demonstrated good postoperative recovery with no significant differences, indicating that the new technology is not inferior to traditional surgical techniques regarding posttraumatic arthritis, limb function, reduction outcomes, or quality of life. Our findings are consistent with those reported by *Madhuchandra* et al.^33^, who applied NPWT to wound management in acute fasciotomy for compartment syndrome and achieved good results in terms of WOMAC scores, limb function, and reduction outcomes.

Some limitations of this study should be noted. First, possible intra-articular lesions were not assessed due to incomplete MRI data. Second, we used X-rays, which are less effective than CT scans, to evaluate the reduction efficacy; however, performing CT scans at every follow-up is impractical. Last, and most importantly, this study is retrospective with a small sample size. The follow-up period of one year is limited compared to the more commonly used five-year follow-up. Future large-sample, multicentre prospective studies and meta-analyses are necessary to draw definitive conclusions about the efficacy of this technique.

## Conclusion

In summary, surgery after the swelling subsides is the generally accepted approach for treating TPFs with limb swelling. However, if deswelling can be accelerated and soft tissue-related complications can be avoided, surgery during the acute swelling phase becomes feasible. This study demonstrated that this new technique yields satisfactory results in treating TPFs with limb swelling. The advantage of this novel approach is that it changes the surgical indications for these patients, shortens the preoperative waiting time and treatment duration, reduces the incidence of perioperative complications, and has good clinical and radiological outcomes.

## Electronic supplementary material

Below is the link to the electronic supplementary material.

Supplementary Material 1

## Data Availability

The data are available on a reasonable demand from the corresponding author.

## Abbreviations

TPFs: Tibial plateau fractures
ORIF: Open reduction internal fixation
NPWT: Negative pressure wound therapy
BMI: Body mass index
VAS: Visual analogue scale
WOMAC: Western Ontario and McMaster University Osteoarthritis Index
PF: Physical function
RP: Role-physical
BP: Body pain
GH: General health
VT: Vitality
SF: Social function
RE: Role-emotional
MH: Mental health

## References

[1] Stephens, A. et al. Diagnostic impacts on management of soft tissue injuries associated with tibial plateau fractures: a narrative review. *Injury* **55**(6), 111546 (2024). 38599010 10.1016/j.injury.2024.111546

[2] Cuéllar-Avaroma, A., King-Martínez, A. C., Hernández-Salgado, A. & Torres-González, R. [Complications in complex fractures of the tibial plateau and associated factors]. *Cir. Cir.* **74**(5), 351–357 (2006). 17224106

[3] van Dreumel, R. L., van Wunnik, B. P., Janssen, L., Simons, P. C. & Janzing, H. M. Mid- to long-term functional outcome after open reduction and internal fixation of tibial plateau fractures. *Injury* **46**(8), 1608–1612 (2015). 26071324 10.1016/j.injury.2015.05.035

[4] Egol, K. A., Tejwani, N. C., Capla, E. L., Wolinsky, P. L. & Koval, K. J. Staged management of high-energy proximal tibia fractures (OTA types 41): the results of a prospective, standardized protocol. *J. Orthop. Trauma.* **19**(7), 448–455 (2005). discussion 456. 16056075 10.1097/01.bot.0000171881.11205.80

[5] Parekh, A. A. et al. Treatment of distal femur and proximal tibia fractures with external fixation followed by planned conversion to internal fixation. *J. Trauma.* **64**(3), 736–739 (2008). 18332816 10.1097/TA.0b013e31804d492b

[6] Frosch, K. H., Korthaus, A., Thiesen, D., Frings, J. & Krause, M. The concept of direct approach to lateral tibial plateau fractures and stepwise extension as needed. *Eur. J. Trauma. Emerg. Surg.* **46**(6), 1211–1219 (2020). 32607776 10.1007/s00068-020-01422-0 PMC7691307

[7] Adams, J. D. J. Jr. & Loeffler, M. F. Soft tissue Injury considerations in the treatment of Tibial Plateau fractures. *Orthop. Clin. North. Am.* **51**(4), 471–479 (2020). 32950216 10.1016/j.ocl.2020.06.003

[8] Wang, T., Guo, J., Long, Y. & Hou, Z. Predictors of acute compartment syndrome in patients with tibial fractures: a meta-analysis. *Int. Orthop.* **47**(1), 51–65 (2023). 36450888 10.1007/s00264-022-05643-3

[9] Giordano, C. P. & Koval, K. J. Treatment of fracture blisters: a prospective study of 53 cases. *J. Orthop. Trauma.* **9**(2), 171–176 (1995). 7776039 10.1097/00005131-199504000-00014

[10] Giordano, C. P., Koval, K. J., Zuckerman, J. D. & Desai, P. Fracture blisters. *Clin. Orthop. Relat. Res.* **307**, 214–221 (1994). 7924035

[11] Barwar, N., Elhence, A., Banerjee, S. & Gahlot, N. Does a staged treatment of high energy tibial plateau fractures affect functional results and bony union? A case series. *Chin. J. Traumatol.* **23**(4), 238–242 (2020). 32249025 10.1016/j.cjtee.2020.03.002 PMC7451683

[12] Sood, L. et al. Compartment syndrome-early diagnosis and treatment. *Indian J. Orthop.* **35**(03), 177–179 (2001).

[13] Kandemir, U. & Maclean, J. Surgical approaches for tibial plateau fractures. *J. Knee Surg.* **27**(1), 21–29 (2014). 24357044 10.1055/s-0033-1363519

[14] dos Santos, L. M., Stewart, G., Meert, K. & Rosenberg, N. M. Soft tissue swelling with fractures: abuse versus nonintentional. *Pediatr. Emerg. Care*. **11**(4), 215–216 (1995). 8532564 10.1097/00006565-199508000-00005

[15] Venturi, M. L., Attinger, C. E., Mesbahi, A. N., Hess, C. L. & Graw, K. S. Mechanisms and clinical applications of the vacuum-assisted closure (VAC) device: a review. *Am. J. Clin. Dermatol.* **6**(3), 185–194 (2005). 15943495 10.2165/00128071-200506030-00005

[16] Agarwal, P., Kukrele, R. & Sharma, D. Vacuum assisted closure (VAC)/negative pressure wound therapy (NPWT) for difficult wounds: a review. *J. Clin. Orthop. Trauma.* **10**(5), 845–848 (2019). 31528055 10.1016/j.jcot.2019.06.015 PMC6739293

[17] Gage, M. J., Yoon, R. S., Egol, K. A. & Liporace, F. A. Uses of negative pressure wound therapy in orthopedic trauma. *Orthop. Clin. North. Am.* **46**(2), 227–234 (2015). 25771317 10.1016/j.ocl.2014.11.002

[18] Stevens, D. G., Beharry, R., McKee, M. D., Waddell, J. P. & Schemitsch, E. H. The long-term functional outcome of operatively treated tibial plateau fractures. *J. Orthop. Trauma.* **15**(5), 312–320 (2001). 11433134 10.1097/00005131-200106000-00002

[19] Sun, L., Qin, S., Pan, Z., Sun, L. & Xing, W. Homeopathic ankle dislocation for treatment of unstable trimalleolar fractures involving posterior die-punch fragment: a retrospective cohort study. *Orthop. Surg.* **16**(5), 1230–1238 (2024). 38556478 10.1111/os.14047 PMC11062885

[20] Ali, A. M. et al. Influence of bone quality on the strength of internal and external fixation of tibial plateau fractures. *J. Orthop. Res.* **24**(11), 2080–2086 (2006). 16944472 10.1002/jor.20270

[21] Stannard, J. P., Wilson, T. C., Volgas, D. A. & Alonso, J. E. The less invasive stabilization system in the treatment of complex fractures of the tibial plateau: short-term results. *J. Orthop. Trauma.* **18**(8), 552–558 (2004). 15475852 10.1097/00005131-200409000-00012

[22] Dirschl, D. R. & Del Gaizo, D. Staged management of tibial plateau fractures. *Am. J. Orthop. (Belle Mead NJ)*. **36**(4 Suppl), 12–17 (2007). 17547353

[23] Vendeuvre, T. et al. Comparative evaluation of minimally invasive ‘tibial tuberoplasty’ surgical technique versus conventional open surgery for Schatzker II-III tibial plateau fractures: design of a multicentre, randomised, controlled and blinded trial (TUBERIMPACT study). *BMJ Open.* **9**(8), e026962 (2019). 31481365 10.1136/bmjopen-2018-026962 PMC6731842

[24] Wang, J. Q. et al. Arthroscopic-assisted balloon tibioplasty versus open reduction internal fixation (ORIF) for treatment of Schatzker II-IV tibial plateau fractures: study protocol of a randomised controlled trial. *BMJ Open.* **8**(8), e021667 (2018). 30093519 10.1136/bmjopen-2018-021667 PMC6089321

[25] Raza, H., Hashmi, P., Abbas, K. & Hafeez, K. Minimally invasive plate osteosynthesis for tibial plateau fractures. *J. Orthop. Surg. (Hong Kong)*. **20**(1), 42–47 (2012). 22535810 10.1177/230949901202000109

[26] Barei, D. P., Nork, S. E., Mills, W. J., Henley, M. B. & Benirschke, S. K. Complications associated with internal fixation of high-energy bicondylar tibial plateau fractures utilizing a two-incision technique. *J. Orthop. Trauma.* **18**(10), 649–657 (2004). 15507817 10.1097/00005131-200411000-00001

[27] Matsumura, T., Nakashima, M., Takahashi, T. & Takeshita, K. Clinical outcomes of open reduction and internal fixation for intra-articular complex tibial plateau non-union with 3-year minimum follow-up. *J. Orthop. Sci.* **26**(3), 403–408 (2021). 32389354 10.1016/j.jos.2020.04.003

[28] Szczesny, G., Olszewski, W. L. & Deszczyński, J. [Post-traumatic lymphatic and venous drainage changes in persistent edema of lower extremities]. *Chir. Narzadow Ruchu Ortop. Pol.* **65**(3), 315–325 (2000). 11057020

[29] Tu, Y. K., On Tong, G., Wu, C. H., Sananpanich, K. & Kakinoki, R. Soft-tissue injury in orthopaedic trauma. *Injury* **39**(Suppl 4), 3–17 (2008). 18804581 10.1016/j.injury.2008.08.027

[30] Yaremchuk, M. J. & Gan, B. S. Soft tissue management of open tibia fractures. *Acta Orthop. Belg.* **62**(Suppl 1), 188–192 (1996). 9084568

[31] Südkamp, N. P. Soft-tissue injury: pathophysiology and its influence on fracture management. *AO Principles of Fracture Management Stuttgart* 59–77 (Thieme, 2000).

[32] Bosse, M. J. et al. A prospective evaluation of the clinical utility of the lower-extremity injury-severity scores. *J. Bone Joint Surg. Am.* **83**(1), 3–14 (2001). 11205855 10.2106/00004623-200101000-00002

[33] Madhuchandra, P. & Muthukumar Balaji, S. A novel approach in the management of Tibial Plateau fractures with compartment syndrome. *Indian J. Orthop.* **57**(9), 1435–1442 (2023). 37609025 10.1007/s43465-023-00955-x PMC10441992

[34] Kakagia, D. et al. Wound closure of leg fasciotomy: comparison of vacuum-assisted closure versus shoelace technique. A randomised study. *Injury* **45**(5), 890–893 (2014). 22377275 10.1016/j.injury.2012.02.002

[35] Kuenlen, A. et al. Influence of VAC therapy on perfusion and edema of gracilis flaps: prospective case-control study. *Plast. Reconstr. Surg. Glob Open.* **11**(4), e4964 (2023). 37124381 10.1097/GOX.0000000000004964 PMC10145892

[36] Aydin, U., Gorur, A., Findik, O., Yildirim, A. & Kocogullari, C. U. Therapeutic efficacy of vacuum-assisted-closure therapy in the treatment of lymphatic complications following peripheral vascular interventions and surgeries. *Vascular* **23**(1), 41–46 (2015). 24676535 10.1177/1708538114529950

[37] Normandin, S. et al. Negative pressure wound therapy: mechanism of action and clinical applications. *Semin Plast. Surg.* **35**(3), 164–170 (2021). 34526864 10.1055/s-0041-1731792 PMC8432996

[38] Glass, G. E., Murphy, G. F., Esmaeili, A., Lai, L. M. & Nanchahal, J. Systematic review of molecular mechanism of action of negative-pressure wound therapy. *Br. J. Surg.* **101**(13), 1627–1636 (2014). 25294112 10.1002/bjs.9636

[39] Ravindhran, B. et al. Molecular mechanisms of action of negative pressure wound therapy: a systematic review. *Expert Rev. Mol. Med.* **25**, e29 (2023). 37853784 10.1017/erm.2023.24

[40] O’Sullivan, O. et al. Current status of catabolic, anabolic and inflammatory biomarkers associated with structural and symptomatic changes in the chronic phase of post-traumatic knee osteoarthritis- a systematic review. *Osteoarthr. Cartil. Open.* **5**(4), 100412 (2023). 37877037 10.1016/j.ocarto.2023.100412 PMC10590857

[41] Zhao, R. et al. Inflammatory factors are crucial for the pathogenesis of post-traumatic osteoarthritis confirmed by a novel porcine model: idealized anterior cruciate ligament reconstruction and gait analysis. *Int. Immunopharmacol.* **99**, 107905 (2021). 34242997 10.1016/j.intimp.2021.107905

[42] Furman, B. D. et al. Targeting pro-inflammatory cytokines following joint injury: acute intra-articular inhibition of interleukin-1 following knee injury prevents post-traumatic arthritis. *Arthritis Res. Ther.* **16**(3), R134 (2014). 24964765 10.1186/ar4591 PMC4229982

[43] Kilpadi, D. V. et al. Effect of vacuum assisted closure therapy on early systemic cytokine levels in a swine model. *Wound Repair. Regen.* **14**(2), 210–215 (2006). 16630111 10.1111/j.1743-6109.2006.00112.x

