# Supplementary material for: A novel approach for managing the incisions of tibial plateau fractures with soft tissue swelling
Source: Sci Rep. 2025 Jan 21;15:2683. doi: 10.1038/s41598-025-86125-5 (PMC11751073; doi:10.1038/s41598-025-86125-5)
Supplement: Supplementary file 1 — Supplementary Material 1 [file 41598_2025_86125_MOESM1_ESM.docx]

**Supplementary Table 1 Swelling Severity Assessment Criteria**

| **Grade** | **Clinical Presentation** |
| --- | --- |
| **Ⅰ** | Mild swelling with visible skin markings |
| **Ⅱ** | Significant swelling with obliteration of skin markings, but no blisters |
| **Ⅲ** | The skin is hard and taut, with serous blisters. |
| **Ⅳ** | Bloody blisters or compartment syndrome. |

**Supplementary Table 2 Modified Rasmussen functional scoring system**

|  | ***Points*** |
| --- | --- |
| **Pain** |  |
| **None** | 6 |
| **Occasional** | 5 |
| **Stabbing pain in certain positioned** | 3 |
| **Constant pain after activity** | 1 |
| **Significant rest pain** | -3 |
| **Walking capacity** |  |
| **Normal walking capacity for age** | 6 |
| **Walking outdoor more than one hour** | 5 |
| **Walking outdoor 15 min–1 h** | 3 |
| **Walking ourdoor < 15 min** | 1 |
| **Walking indoor only** | 0 |
| **Wheel chair or bed ridden** | -3 |
| **Knee extension** |  |
| **Normal** | 4 |
| **Lack of extension < 10°** | 2 |
| **Lack of extension > 10°** | 0 |
| **Lack of extension > 20°** | -2 |
| **Total range of motion** |  |
| **Full** | 6 |
| **Atleast 120°** | 5 |
| **Atleast 90°** | 3 |
| **Atleast 60°** | 1 |
| **< 60°** | -3 |
| **Stability** |  |
| **Normal stability in extension and 20° flexion** | 6 |
| **Abnormal in stability in 20° flexion** | 4 |
| **Instability in extension < 10°** | 2 |
| **Instability in extension > 10°** | 0 |
| **Power of quadriceps** |  |
| **Grade 5** | 2 |
| **Grade 3–4** | 1 |
| **Grade < 3** | -2 |
| **Maximum score** | 30 |
| **Excellent** | 28–30 |
| **Good** | 24–27 |
| **Fair** | 20–23 |
| **Poor** | < 20 |

**Supplementary Table 3 Modified Rasmussen Radiological scoring system**

| ***X ray findings*** | ***Points*** |
| --- | --- |
| **Articular depressions** |  |
| **None** | 3 |
| **< 5 mm** | 2 |
| **6–10 mm** | 1 |
| **> 10 mm** | 0 |
| **Condylar widening** |  |
| **None** | 3 |
| **< 5 mm** | 2 |
| **6–10 mm** | 1 |
| **>10 mm** | 0 |
| **Valgus/varus angulation** |  |
| **None** | 3 |
| **< 10 mm** | 2 |
| **10–20 mm** | 1 |
| **>20 mm** | 0 |
| **Osteoarthrosis** |  |
| **None/no progress** | 1 |
| **Progression by 1 grade** | 0 |
| **Progression by > 1 grade** | -1 |
| **Maximum Score** | 10 |
| **Excellent** | 9–10 |
| **Good** | 7–8 |
| **Fair** | 5–6 |
| **Poor** | < 5 |
